# Plasmablastic Lymphoma in an Immunocompetent Patient with MDS/MPN with Ring Sideroblasts and Thrombocytosis—A Case Report

**DOI:** 10.1155/2018/2525070

**Published:** 2018-11-04

**Authors:** Anthi Bouchla, Sotirios G. Papageorgiou, Zoi Tsakiraki, Eirini Glezou, George Pavlidis, Georgia Stavroulaki, Efthimia Bazani, Periklis Foukas, Vasiliki Pappa

**Affiliations:** ^1^Second Department of Internal Medicine and Research Unit, University General Hospital “Attikon”, 1 Rimini St., Haidari, 12462 Athens, Greece; ^2^2nd Department of Pathology, National and Kapodistrian University of Athens, Attikon University Hospital, Athens, Greece

## Abstract

Plasmablastic lymphoma (PBL) is a rare, aggressive type of B-cell non-Hodgkin lymphoma with the vast majority of patients responding poorly to treatment or progressing shortly thereafter. Cyclophosphamide-doxorubicin-vincristine-prednisolone (CHOP) or CHOP-like regimens have disappointing results in this setting. We report a case of PBL arising in a previously diagnosed myelodysplastic/myeloproliferative (MDS/MPN) with ring sideroblasts and thrombocytopenia (RS-T), HIV-negative patient treated with the combination of CHOP and bortezomib. The patient achieved complete metabolic response, which has lasted one year, longer by far than would have been expected with the sole use of CHOP.

## 1. Introduction

Plasmablastic lymphoma (PBL) is a subtype of aggressive B-cell non-Hodgkin lymphoma (NHL) that was recently recognized as a distinct entity. In the initial report by Delecluse et al., most patients were HIV seropositive with a large B-cell NHL of the oral cavity with unique immunohistological features, mainly absence of the CD20 expression, stead expression of VS38c, and variable expression of CD79a [1].

The World Health Organization (WHO) classification defines PBL as an aggressive B-cell NHL which is characterized by diffuse proliferation of large neoplastic cells, resembling B immunoblasts in which all tumor cells have the immunophenotype of plasma cells [2, 3]. During the last years, several case reports and small series have been reported in both immunodeficient and immunocompetent patients and involving various anatomic sites [4–6]. However, PBL remains a rare disease that has not been fully depicted, a diagnostic challenge due to its similarities with multiple myeloma (MM), and a therapeutic challenge since no standard of care exists and prognosis is poor.

Refractory anemia with ring sideroblasts associated with marked thrombocytosis (RARS-T), which was a provisional entity within the myelodysplastic syndromes/myeloproliferative neoplasms (MDS/MPN) unclassifiable group in the WHO 2008 classification, has now been accepted as a distinct entity in the revised WHO 2016 classification [2, 3]. Currently, the disease is termed as MDS/MPN with ring sideroblasts and thrombocytosis (MDS/MPN with RS-T), and the diagnostic criteria are summarized in Table 1 [7].

The coexistence of various plasma cell dyscrasias with different types of myeloid neoplasms usually occurs in patients who received long-term chemotherapy with alkylating agents prior to the development of leukemia. Only rare cases of simultaneous coexistence of these two malignancies unrelated to prior therapy have been reported [8–11]. However, the coexistence of PBL with myeloid neoplasms has not been described to date. To our knowledge, we report the unique case of PBL arising in the setting of a previously diagnosed MDS/MPN with RS-T treated only with erythropoietin alpha in an immunocompetent and HIV-negative patient.

## 2. Case Report

A 74- year-old Caucasian male was referred to our hematology department in November 2016 for hypochromic microcytic anemia requiring red blood cell (RBC) transfusions. He was known to carry a beta-thalassemic gene mutation, but his hemoglobin levels had dropped gradually to 5.9 g/dL in the last year with no apparent gastrointestinal blood loss. His medical history included smoking, arterial hypertension, and a thoracic aneurysm of 46 mm wide and an abdominal aneurysm of 30 mm wide with no history of coronary arterial disease. He was currently on metoprolol 25 mg per day.

Upon referral, the patient had already been transfused with 3 units of red blood cells, and his blood counts were white blood count (WBC): 5.26 × 10^3^/*μ*L, red blood count (RBC): 3.97 × 10^3^/*μ*L, hematocrit (HCT): 31.4%, hemoglobin (Hb): 9.2 g/dL, mean corpuscular volume (MCV): 79.2 fl, mean corpuscular hemoglobin concentration (MCHC): 23.2 g/dL, and platelets (PLT): 507 × 10^3^/*μ*L.

The bone marrow smear revealed hypercellularity with dyserythropoiesis and increased megakaryocytes with no excess blasts. The iron stain showed dense iron deposits with ring sideroblasts >15% of erythroblasts. Cytogenetic analysis revealed normal karyotype. The BCR-ABL1 fusion genes, and rearrangements of PDGFRA and PDGFRB, were negative. Similar the JAK2-V617F mutation was not detected. The patient was diagnosed with RARS-T according to WHO 2008 or MDS/MPN with RS-T according to WHO 2016, and he was started on erythropoetin alpha, 40,000 units per week administered subcutaneous (s.c.) and acetylsalicylic acid 100 mg per day. He soon became transfusion independent.

**Figure 1:**
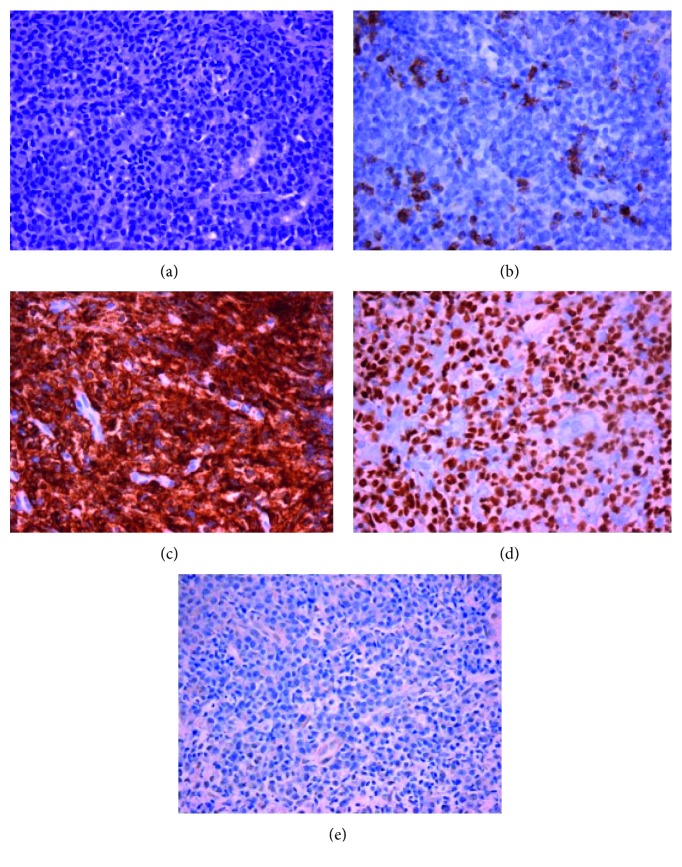
(a) Large atypical lymphoid tumor cells with plasmacytoid morphology, abundant cytoplasm, and eccentric large nuclei with vesicular chromatin, some of which demonstrate nucleoli (H&E 40x). (b) Tumor cells were negative for CD20 antigen. Some positive reactive B lymphocytes can be discerned. Intense, uniform immunoreactivity for plasmacytoid differentiation-associated antigens CD138 (c) and MUM-1 (d). (e) EBER in situ hybridization was negative.

Nine months later, in August 2018, he sought medical advice for a right submandicular mass that had been rapidly growing for the past five days. The patient was afebrile and in good performance status (PS) (ECOG PS = 1). His WBC count was 7.7 × 10^6^/*μ*L, with 53% neutrophils; his C-reactive protein (CRP) and erythrocyte sedimentation rate (ESR) were elevated (32.3 mg/L and 120 mm, respectively). The biochemistry panel was in normal ranges except for elevated lactate dehydrogenase (LDH): 257 U/L (normal range: 135–225 U/L). A computer tomography (CT) scan of the neck showed a right submandicular lymph node block measuring 5.5 × 3.2 cm with focal cystic degeneration with peripheral contrast media attenuation. The lesion was regarded as lymph node abscess, and the patient was admitted to the otorhinolaryngological department where he was empirically started on intravenous ciprofloxacin and clindamycin with no remission of the lesion. Subsequently, an ultrasound-guided fine needle biopsy was performed, and the microscopy revealed a diffuse infiltrate of large neoplastic lymphoid cells in a cohesive pattern with plasmablastic and plasmacytic features, containing eccentric nuclei with vesicular chromatin, abundant cytoplasm, and prominent central nucleolus in some of them. Some small mature tumor cells with plasmacytic differentiation were identified. Concomitant neoplastic necrosis and histiocytic/neutrophilic infiltration was noted. An immunohistochemical study was performed which revealed negativity for B-cell markers CD20, Pax-5, and only weak, focal expression of CD79a. Plasmacytoid differentiation markers CD38, CD138, MUM-1, and EMA were uniformly, intensely positive. The proliferation index demonstrated by Ki-67 expression was approximately 90%. Epstein–Barr virus was not detected by the means of EBER in situ hybridization. MYC expression was not assessed (Figure 1). A repeated bone marrow biopsy showed red cell dysplasia with RS > 20% of erythroblasts and absence of plasma cells, while immunoelectrophoresis showed diffuse elevation of gamma globulins and immunofixation was normal. Testing for HIV1, 2 antibodies were negative. A diagnosis of PBL was made.

A CT scan-staging approach revealed no lymph enlargement besides the right submandicular lymph node block; however, the 18-fluorodeoxyglucose- (18-FDG-) positron emission tomography (PET)-CT scan revealed an increased uptake in the base of the tongue (SUV max. = 5.8), in addition to an increased uptake in the submandicular lymph node block (SUV max. = 4.5) and in a right cervical lymph node (SUV max. = 3.1). His cerebrospinal fluid (CSF) analysis was normal. Based on the Ann Arbor staging system, the patient was staged as IIE, and according to the international prognostic index (IPI) score, he had high intermediate (IPI: 2). He was started on CHOP every 21 days plus bortezomib 1.3 mg/m^2^ was administered s.c. on days 1, 4, 8, and 11 of every 21-day cycle and central nervous system (CNS) prophylaxis with intrathecal methotrexate at a dose 12.5 mg on day 1. Significant clinical improvement was noted by the completion of the first cycle with minimal palpable residual mass. After 6 cycles of therapy, he was on complete metabolic remission with negative 18-FDG-PET/CT scan and at 12 months' follow-up he was still in complete remission, with negative CT scans.

## 3. Discussion

PBL is a rare and aggressive disease. Its greatest incidence is found in HIV-positive individuals who also present an EBV coinfection. It may also be associated with other immunodeficiency conditions, including immunosuppresion for autoimmune disease and advanced age. The median age at presentation is 50 years, with a broad distribution but mainly affecting adults. PBL presents most frequently as a mass in the oral cavity, but it is also encountered in other extranodal sites, particularly mucosal sites. Nodal involvement is not common, but it is more frequent in HIV-negative patients. Most patients present at an advanced stage (III or IV) [12, 13]. The prognosis is poor with a median OS of 11 months in HIV-negative immunocompetent patients and 10 months in HIV-positive patients [14]. Our patient was 74 years old, immunocompetent and had a mucosal and a nodal site of involvement. He was at an early stage at presentation. In addition, he had already been diagnosed with MDS/MPN RS-T nine months before PBL diagnosis.

To our knowledge, this is the first report of a patient with MDS/MPN RS-T who developed PBL. This raises the question whether there is an association between myelodysplasia and PBL or it is only a matter of the patient's advanced age. In low-risk myelodysplastic syndromes (MDS), the immune system is dysregulated causing increased apoptosis of the normal hematopoietic precursors [15], but no direct association between MDS and immunosuppression has been reported. However, a causal relationship between the patient's MDS and the emergence of PBL cannot be ruled out. Ideally, the best way to prove the association between myelodysplasia and PBL would have been molecular analysis of both malignant clones using next generation sequencing; however, this was not feasible in our case.

Given the rarity and heterogeneity of PBL, no large randomized controlled therapeutic studies have been conducted so far. Treatment guidelines have been published for HIV-affected cases with PBL, which include dose-adjusted EPOCH, CODOX-M/IVAC, and hyper-CVAD, as CHOP is not considered adequate [16]. However, two studies of patients with PBL treated with chemotherapy regimens more intensive than CHOP did not identify a survival benefit [17, 18]. Intensive regimens were considered too toxic for our patient due to his old age and concomitant myelodysplasia.

The use of antimyeloma agents has been based on the fact of the plasmacytic differentiation of PBL cells and on the detection of common genetic abnormalities between ΜΜ undergoing blastic transformation and PBL [19] although the experience is limited to case reports. The proteasome inhibitor bortezomib constitutes the cornerstone of frontline treatment in MM achieving rapid responses, protecting kidney function, and overcoming the adverse prognosis of poor cytogenetics. In addition, bortezomib has been shown to be effective in patients with post-germinal center (GC) diffuse large B-cell lymphoma (DLBCL), inducing higher response and survival rates when used in combination with anthracycline-containing regimens [19] and has been shown promising in PBL patients in combination with CHOP or CHOP-like regimens both in HIV-positive [9] and in HIV-negative patients [20]. As a result, the combination of CHOP plus bortezomib appeared a suitable choice for our patient. The patient's risk of CNS relapse was intermediate according to the CNS-IPI score [21]; however; we decided to administer CNS prophylaxis with intrathecal methotrexate due to the extranodal involvement and the high proliferation rate as expressed by the Ki-67 index.

Autologous hematopoietic stem cell transplantation (auto-HSCT) in first remission seems to benefit PBL patients. The International Blood and Marrow Transplant Research (IBMTR) reported on eleven PBL patients who underwent transplantation in the first complete remission. Relapse data were not reported, but 1-year and 3-year OS rates were 69% (95% confidence interval [CI], 48% to 87%) and 45% (95% CI, 21% to 71%), respectively [22]. Unfortunately, our patient was not eligible for auto-HSCT because of old age.

In conclusion, our case is remarkable for the coexistence of PBL with MDS/MPN with RS-T. Furthermore, the combination of CHOP and bortezomib seems to have benefited the patient far more than what would have been expected with the sole use of CHOP, as shown by his long (almost one year) event-free survival.

## Figures and Tables

**Table 1:** Diagnostic criteria for MDS/MPN with RS-T according to WHO 2016 classification [3].

Diagnostic criteria
(i) Anemia associated with erythroid lineage dysplasia with or without multilineage dysplasia ≥15% sideroblasts, ^*∗*^<1% in PB, and <5% blasts in BM
(ii) Persistent thrombocytosis with platelet count ≥450 × 10^9^/L
(iii) Presence of SF3B1 mutation or in the absence of SF3B1 mutation, no history of recent cytotoxic or growth factor therapy that could explain the myelodysplastic/myeloproliferative features^†^
(iv) No BCR-ABL1 fusion gene, no rearrangement of PDGFRA, PDGFRB, or FGFR1; PCM1-JAK2, no t(3;3) (q21; q26), inv(3) (q12;q26) or del(5q)^‡^
(v) No preceding history of MPN, MDS (except MDS-RS), or other type MDS/MPN

^*∗*^At least 15% ring sideroblasts required even if SF3B1 mutation is detected. ^†^A diagnosis of MDS/MPN-RS-T is strongly supported by the presence of SF3B1 mutation together with a mutation in JAK2 V617F, CALR, or MPL genes. ^‡^In a case which otherwise fulfills the diagnostic criteria for MDS with isolated del(5q-), no or minimal absolute basophilia, basophils usually 2% of leukocytes.
